# Parameter Determination of the 2S2P1D Model and Havriliak–Negami Model Based on the Genetic Algorithm and Levenberg–Marquardt Optimization Algorithm

**DOI:** 10.3390/polym15112540

**Published:** 2023-05-31

**Authors:** Mingzhu Qiu, Peng Cao, Liang Cao, Zhifei Tan, Chuantao Hou, Long Wang, Jianru Wang

**Affiliations:** 1 Faculty of Architecture, Civil and Transportation Engineering, Beijing University of Technology, Beijing 100084, China; qiumingzhu@emails.bjut.edu.cn (M.Q.);; 2 Department of Civil and Environmental Engineering, The Hong Kong Polytechnic University, Hong Kong SAR, China; zhi-fei.tan@connect.polyu.hk; 3 Science and Technology on Reliability and Environmental Engineering Laboratory, Beijing Institute of Structure and Environment Engineering, Beijing 100076, China; houcht@126.com (C.H.); longwangfr@126.com (L.W.); 4 The 41st Institute of the Fourth Research Academy of CASC, Xi’an 100124, China

**Keywords:** viscoelastic, genetic algorithm (GA), Levenberg–Marquardt algorithm, 2S2P1D model, Havriliak–Negami model

## Abstract

This study utilizes the genetic algorithm (GA) and Levenberg–Marquardt (L–M) algorithm to optimize the parameter acquisition process for two commonly used viscoelastic models: 2S2P1D and Havriliak–Negami (H–N). The effects of the various combinations of the optimization algorithms on the accuracy of the parameter acquisition in these two constitutive equations are investigated. Furthermore, the applicability of the GA among different viscoelastic constitutive models is analyzed and summarized. The results indicate that the GA can ensure a correlation coefficient of 0.99 between the fitting result and the experimental data of the 2S2P1D model parameters, and it is further proved that the fitting accuracy can be achieved through the secondary optimization via the L–M algorithm. Since the H–N model involves fractional power functions, high-precision fitting by directly fitting the parameters to experimental data is challenging. This study proposes an improved semi-analytical method that first fits the Cole–Cole curve of the H–N model, followed by optimizing the parameters of the H–N model using the GA. The correlation coefficient of the fitting result can be improved to over 0.98. This study also reveals a close relationship between the optimization of the H–N model and the discreteness and overlap of experimental data, which may be attributed to the inclusion of fractional power functions in the H–N model.

## 1. Introduction

Polymers are widely used in various fields, such as construction materials, electronic devices, high-energy explosives, textiles, and medical applications due to the fact of their excellent mechanical properties, including high deformability, damping characteristics, and strong adhesion to other materials [1,2,3,4]. Since World War II, polymeric materials have come to occupy a position of equal importance to metallic materials in the modern industrial system.

In general, polymers exhibit significant temperature- and time-dependent viscoelastic behavior, which is determined by the molecular structure of polymer compounds. Unlike metallic materials, polymers are formed by the polymerization of many small molecules into large polymers, resulting in an interconnected network structure. The viscoelasticity of polymer materials is caused by the deformation of the chain-like polymers under stress. With the widespread use of polymer materials in various engineering fields, an accurate assessment of the viscoelastic properties of polymers has become crucial for predicting their mechanical behavior [5,6].

To accurately describe the viscoelastic response of polymer materials, various constitutive models have been proposed [7]. Common viscoelastic models include the Maxwell model, Kelvin–Voigt model, generalized Maxwell model, standard linear model (SLM), 2S2P1D model, and Huet model [8]. Among these, the 2S2P1D model is a generalized model derived from the Huet–Sayegh model that accurately describes the rheological properties of adhesives and asphalt mixtures [9]. The Havriliak–Negami (H–N) model is of great value in predicting the viscoelastic properties of rubber-like materials and their engineering applications due to the fact of its simplicity and accurate prediction capabilities [10].

Different viscoelastic constitutive models require the determination of different types of viscoelastic parameters. Since viscoelastic constitutive models involve time information, it is difficult to express their expressions directly. They are usually represented using a discretization method. Common discretization methods for viscoelastic constitutive model parameters include Prony series models and Schapery constitutive models [11,12]. Since the parameters of viscoelastic constitutive models are difficult to measure directly, they need to be determined by comparing optimization methods with simulation results [13,14,15,16]. Since Prony series and Schapery constitutive models have a large research base and open source programs for solving the time-varying behavior of polymer materials and structures in complex states in combination with finite element methods, they are widely used for solving the time-dependent behavior of complex states in polymer materials and structures at present [17,18,19,20]. However, the description of the viscoelastic behavior of materials is usually achieved by using various functions, such as exponential, sigmoid functions, and various Bessel functions, to define continuously varying elastic moduli for different loading periods, which are mathematically represented in the form of an integral equation with a graded integral kernel. Mathematically, this is represented by an integral equation containing a series integral kernel [21,22,23,24]. In practical applications, the integral equation needs to be discretized, which means discretizing the continuous exponential function into Prony series. The number of sampling points in the discretization process determines the computational accuracy, and the number of sampling points in the Prony series determines the number of parameters in the constitutive model. Having too many sampling points obviously increases the difficulty of determining the parameters and may lead to overfitting issues [25,26,27,28]. To solve this problem, extensive research has been conducted over the past two decades on methods related to the identification and acquisition of viscoelastic parameters using various optimization algorithms, including gradient-based batch gradient descent, stochastic gradient descent, mini-batch gradient descent, and the Levenberg–Marquardt (L–M) algorithm, as well as gradient-free optimization algorithms, such as genetic algorithm (GA), particle swarm optimization, and neural network optimization algorithms. There are two common methods for obtaining viscoelastic parameters in polymer materials: one is the fitting method, based on relaxation and creep test results, and the other is the fitting method, based on the master curve of the dynamic modulus. The method based on dynamic modulus testing is favored by researchers due to the fact of its high accuracy and wide frequency range. Although gradient and non-gradient optimization algorithms have been widely used in the inverse analysis of viscoelastic model parameters, there have been few mixed optimization algorithms that combine these two methods for the parameter acquisition and optimization of different materials and viscoelastic constitutive models. The use of the GA combined with the non-gradient optimization algorithm to obtain the initial values of the parameters, as well as utilizing the L–M algorithm, a gradient algorithm, for the further optimization of the parameters, is of great significance for improving the accuracy of viscoelastic constitutive model parameters and studying the influence of viscoelastic materials in complex mechanical time-dependent processes.

Based on this, this paper intended to explore the optimization problem of viscoelastic model parameters using different optimization algorithms. Section 2 of this paper mainly introduces the basic theory of GA and their applications in the parameter acquisition and optimization of the 2S2P1D model and H–N model. Detailed calculation processes for inverting parameters are provided. In Section 3, based on experimental data, two methods of optimization inversion are studied for the 2S2P1D model and H–N model: one is based on empirical parameters, and the other is based on the William, Landel, and Ferry (WLF) equation of relaxation time. Furthermore, based on the preliminary fitting results using the GA, a sequential mixed algorithm using GA+L–M is proposed to further optimize the parameters of the two models, and the influence of the L–M algorithm on the optimization results of the GA is investigated. Finally, the impact of the GA and GA+L–M algorithms on obtaining parameter results in these two commonly used viscoelastic constitutive equations is discussed, and the applicability of the GA and GA+L–M algorithms among different models is summarized.

## 2. Methodology and Optimization Study

In this section, two commonly used viscoelastic models, including the 2S2P1D and H–N models, are applied. The formula of the H–N model contains high-order fractional derivatives, complex numbers, and generalized integrals, which place high demands on the selected upper and lower bounds of parameters. Thus, the direct optimization of the dynamic modulus and phase angle usually results in inaccurate values or even a large discrepancy between the solution and the fitting results due to the fact of inappropriate initial values.

### 2.1. Theory of GA

**Figure 1:**
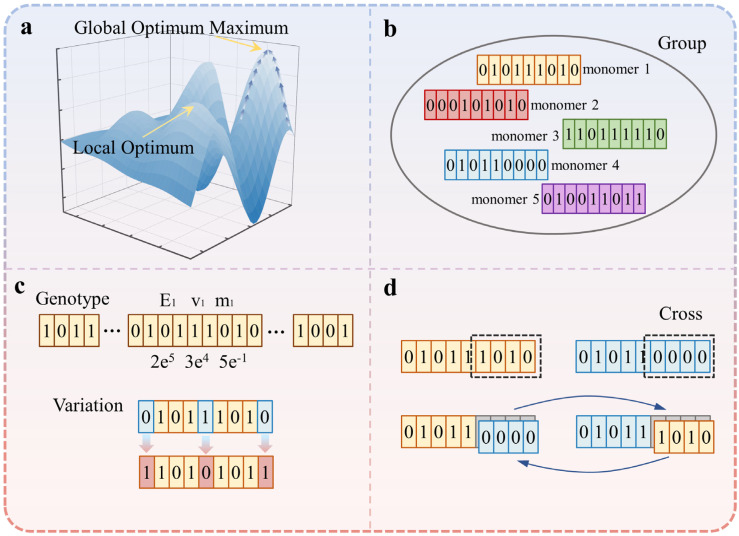
Theory of the genetic algorithm (GA): (**a**) global optimization; (**b**) population; (**c**) genotype and mutation; (**d**) crossover.

Figure 1 illustrates the basic principles of the GA. It is a method that searches for the global optimal solution by simulating the natural evolution process. The algorithm begins with an initial population and utilizes random selection, crossover, and mutation operations to generate individuals that are better adapted to the environment. This iterative process promotes continuous evolution, leading to convergence on a group of individuals highly adapted to the environment, ultimately obtaining the optimal solution to the problem at hand [29]. The fundamental components and operations of the GA include the following: (1) Population—a GA maintains a large population of individuals, representing candidate solutions to the problem. Each individual is represented by a chromosome, and the population can be viewed as a collection of chromosomes. (2) Genotype—in the GA, each individual consists of a chromosome representing a set of genes. (3) Fitness function—at each iteration of the algorithm, individuals are evaluated using a fitness function (also known as an objective function), which quantifies the quality of a solution. (4) Selection—After calculating the fitness of each individual, a selection process determines which individuals should be used to breed and produce the next generation. (5) Crossover—to create new individuals, portions of parent chromosomes from the current generation are exchanged, resulting in two descendant chromosomes. (6) Mutation—The mutation operation simulates gene mutations occurring in the natural genetic environment. It randomly changes the value of a gene with a small probability. In binary encoding, mutation manifests as the random flipping of a gene from 1 to 0 or vice versa. The pseudocode for the GA implemented in this study is provided in Appendix A.

### 2.2. Parameter Optimization of 2S2P1D Model 

#### 2.2.1. Theory of 2S2P1D Model

**Figure 2:**
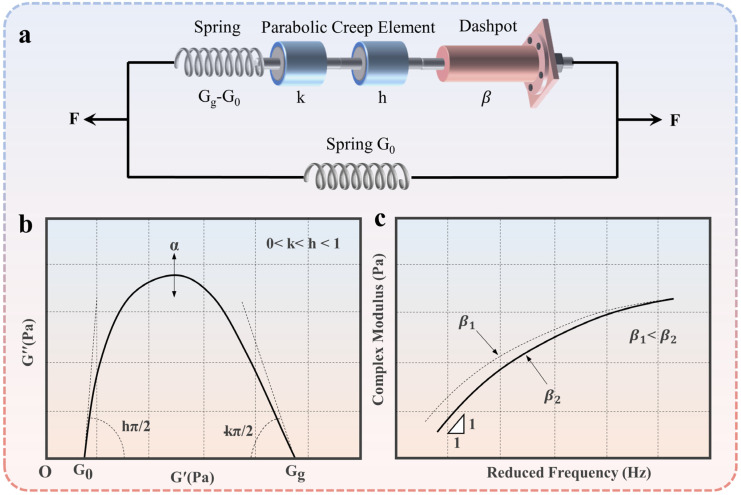
The 2S2P1D model: (**a**) schematic diagram; (**b**) Cole–Cole curve; (**c**) complex modulus master curves.

The 2S2P1D model, initially calibrated at the Ecole Nationale des Travaux Publics de l’Etat (ENTPE) laboratory [30], is a widely used viscoelastic constitutive model for modeling the behavior of viscoelastic materials. “2S2P1D” stands for the abbreviation of two springs, two parabolic creep elements, and one damper. As shown in Figure 2a, the model is an extension of the Huet–Sayegh model. Its basic theory is as follows:

Based on research of Olard and Di Benedetto, as shown in Reference [9], the parabolic creep element obeys the parabolic creep function:

(1) $$J\left(t\right)=a{\left(\frac{t}{\tau }\right)}^{h}$$

The expression of the complex modulus is:

(2) $$G^{*}=\frac{{\left(i\omega \tau \right)}^{h}}{a\Gamma \left(h+1\right)}$$

where $J\left(t\right)$ is the creep function; *h* is the exponential index; 0 < *h* < 1(*h* = 0 (elastic), *h* = 1(viscous)); a is a dimensionless constant; λ is the relaxation time; Γ is the gamma function; *t* is the loading time; τ is the relaxation time (varies only with temperature); *i* is an imaginary number; and ω is the frequency.

For the linear damper, the complex modulus can be expressed as follows:

(3) $$G^{*}=\frac{i\omega \tau }{a}$$

The expression of the 2S2P1D model is shown as follows:

(4) $$G^{*}=G_{0}+\frac{G_{g}-G_{0}}{1+\alpha {\left(i\omega \tau \right)}^{-k}+{\left(i\omega \tau \right)}^{-h}+{\left(i\omega \beta \tau \right)}^{-1}}$$

where *k* and *h* are the exponent indices, with 0 < *k < h <* 1; α is a constant; $G_{0}$ is the static modulus at ω→0; and $G_{g}$ is the glassy modulus at ω→1. As shown in Figure 2b, $G_{0}$ and $G_{g}$ are the intercepts of the Cole–Cole curve on the x-axis. β is a constant. η is defined as:

(5) $$\eta =(G_{g}-G_{0})\beta \tau$$

where η is the Newtonian viscosity, and τ is the relaxation time, which is a function of the temperature, as shown in Figure 2c. Within the temperature range observed in the laboratory, τ can be approximated by the shift factor law based on the WLF equation:

(6) $$\mathrm{lg}\alpha_{T}\left(T\right)=\frac{C_{1}\left(T-T_{r}\right)}{C_{2}+\left(T-T_{r}\right)}$$

(7) $$\tau =a_{T}(T)\times \tau_{0}$$

where *C*_1_ and *C*_2_ are empirical parameters, *T* and *T_r_* are the actual and reference temperatures, $a_{T}$ is the shift factor, and $\tau_{0}$ and $\tau_{i}$ are the relaxation times at temperatures *T_r_* and *T*, respectively. The WLF equation is a significant empirical formula in polymer physics that delineates the correlation between the relaxation time and temperature. In numerous amorphous polymers, the complex modulus at different temperatures can be superimposed into a master curve with a broader frequency range by horizontally shifting the time quantities obtained at different temperatures.

The phase angle can be expressed as:

(8) $$\delta =\tan^{-1}\left(\frac{G^{\prime \prime }}{G^{\prime }}\right)$$

where $G^{\prime }$ and $G^{\prime \prime }$ are the storage and loss moduli. 

From Equation (4), we can see that seven parameters ($G_{0}$, $G_{g}$, k, h, α, β, and τ) are required to be determined in the 2S2P1D model. 

#### 2.2.2. Parameter Optimization Process

In this section, the GA and L–M algorithms are used to conduct the optimization of the 2S2P1D model’s parameters. Two methods (dependent and independent WLF functions) for the optimization are considered. The optimization processes are as follows:

(1) Parameter optimization independent of the WLF function
  In the first approach, the test data obtained at different temperatures are independently optimized, resulting in the determination of the relaxation time ($\tau_{i}$) values for each temperature. Based on Equation (4), we know that six temperature-independent parameters ($G_{0}$, $G_{g}$, k, h, α, and β) and one temperature-dependent parameter ($\tau_{i}$) should be determined. The detailed procedure is given below:
  (1) Take $G_{0}$, $G_{g}$, k, h, α, β, and $\tau_{i}$ as unknowns and the regularized 2-norm sum of the differences between the calculated (based on Equations (4) and (8)) and experimental dynamic moduli and phase angles as the objective function, and then conduct the optimization using the GA considering the given constraints and upper and lower bounds.
  (2) Take the parameters obtained from the GA as the initial values for further optimization using the L–M algorithm.
  (3) Substitute the obtained parameters into Equations (4) and (8) to calculate the corresponding dynamic modulus ($\left|G^{*}\right|$) and phase angle (δ). The correlation coefficient between the calculated values and the original experimental values is calculated to evaluate the fitting results.
(2) Parameter optimization dependent on WLF function
  In the second method, the test data obtained at different temperatures are shifted to the reference temperature (Tr) using the WLF equation, with the subsequent optimization of only one relaxation time value ($\tau_{0}$) at the reference temperature. The specific steps are as follows:
  (1) Take $G_{0}$, $G_{g}$, k, h, α, β, $\tau_{0}$, $C_{1}$, $C_{2}$ and $T_{r}$ as unknowns, and employ the GA to obtain the initial parameter values.
  (2) Take the parameter values obtained with the GA as the initial values and further optimize the parameters using the L–M algorithm.
  (3) Substitute the obtained parameters into Equations (4) and (8) to calculate the corresponding dynamic modulus ($\left|G^{*}\right|$) and phase angle (δ). The correlation coefficient between the calculated values and the original experimental values is calculated to evaluate the fitting results.

### 2.3. Parameter Optimization of the H–N Model

#### 2.3.1. Theory of the H–N Model

The H–N model is widely used to characterize the complex dielectric behavior of polymers. The relationship between the complex modulus and the instantaneous modulus ($E_{0}$) and the long-term modulus ($E_{\infty }$) given by the equation is [10]:

(9) $$E^{*}(\omega )=\frac{E_{0}-E_{\infty }}{{\left[1+{\left(i\omega \tau \right)}^{\alpha }\right]}^{\beta }}+E_{\infty }=E^{\prime }(\omega )+iE^{\prime \prime }(\omega )$$

where ω is the angular frequency; τ is the relaxation time related to temperature; *i* is the unit imaginary number; $E_{0}$, $E_{\infty }$, α, and β are the parameters of the H–N model that are independent of temperature; and $E^{\prime }(\omega )$ and $E^{\prime \prime }(\omega )$ are the storage modulus and loss modulus, respectively. However, the H–N model is highly intricate and involves fractional and nonreal power functions. This complexity can lead to nonreal outcomes if the parameter analysis is directly performed on these functions. To address this concern, this paper employed a semi-analytical method to conduct the inverse analysis on the constitutive model. This approach involves plotting the Cole–Cole curve and utilizing the semi-analytical method to determine the polynomial coefficients. Subsequently, these coefficients are substituted into the Cole–Cole diagram using the four tangent equations, which are presented below:

(10) $$\tan\varphi =\underset{\omega \to 0}{\lim}\frac{dE^{\prime \prime }(\omega )}{dE^{\prime }(\omega )}=\tan\frac{\alpha \pi }{2}$$

(11) $$\tan\theta =\underset{\omega \to \infty }{\lim}\frac{dE^{\prime \prime }(\omega )}{dE^{\prime }(\omega )}=-\tan\frac{\alpha \beta \pi }{2}$$

if $\left|i\omega_{n}\tau \right|<1$,

(12) $$\tan\Phi =\frac{1}{2}\tan\frac{\alpha \pi }{2}+\frac{3}{2}\cot\frac{\alpha \pi }{2}$$

(13) $$\tan\Theta =\left[4\Gamma \left(2+\beta \right)\Gamma \left(\beta \right)/\Gamma^{2}\left(1+\beta \right)-\frac{3}{2}\right]\cot\frac{\alpha \pi }{2}-\frac{1}{2}\tan\frac{\alpha \pi }{2}$$

if $\left|i\omega_{n}\tau \right|>1$,

(14) $$\tan\Phi =\frac{\frac{\Gamma^{\beta }\left(1+\beta \right){\left(1+\beta \right)}^{\beta }}{\Gamma^{\beta }\left(\beta \right)\beta^{\beta }}\cos^{-\beta -1}\frac{\alpha \beta \pi }{2}\cos^{\beta }\frac{\alpha \left(1+\beta \right)\pi }{2}+\beta -1}{\tan\frac{\alpha \beta \pi }{2}-\beta \tan\frac{\alpha \left(1+\beta \right)\pi }{2}}$$

(15) $$\tan\Theta ={\left(\left(1+\beta \right)\tan\frac{\alpha \beta \pi }{2}-\beta \tan\frac{\alpha \left(1+\beta \right)\pi }{2}\right)}^{-1}$$

**Figure 3:**
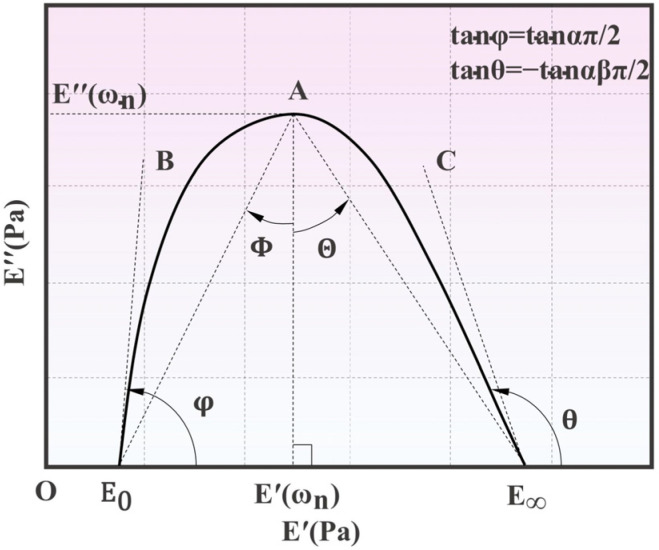
Schematic diagram of the four tangent angles in the Cole-Cole curve of the H-N model.

The temperature-independent parameters of the H–N model, namely, the four angles (φ, θ, Φ, and Θ), can be initially determined based on Equations (10)–(15). In Figure 3, the Cole–Cole diagram is illustrated. Typically, a Cole–Cole diagram exhibits an inverted U-shaped curve for viscoelastic materials. The $OE^{\prime }$ axis is the storage modulus axis, and the $OE^{\prime \prime }$ axis is the loss modulus axis. The curve $E_{0}AE_{\infty }$ is the Cole–Cole curve, which is typically employed to depict the relationship between the loss modulus and storage modulus of viscoelastic materials [10]. 

#### 2.3.2. Parameter Optimization Process

Previous studies have directly optimized the H–N model’s parameters based on the dynamic modulus and phase angle, a method highly sensitive to the optimization range and initial values [10]. Moreover, the H–N model formula incorporates high-order fractional derivatives, complex numbers, and generalized integrals, which impose strict requirements on the selection of the upper and lower parameter bounds. Consequently, the direct optimization of the dynamic modulus and phase angle often yields inaccurate values and may result in substantial discrepancies between the solution and the fitting results due to the fact of inappropriate initial values. In order to solve these issues and ensure numerical stability in the calculation of the complex H–N model, we propose a progressive method. In contrast to the direct optimization approach, the Cole–Cole curve, which describes the relationship between $E^{\prime }$ and $E^{\prime \prime }$, is initially fitted using a third-order polynomial function to determine certain parameter values of the H–N model. Subsequently, the GA is employed to further optimize the parameter values. The detailed optimization process based on the proposed progressive method is outlined below:

(1) Plot the storage modulus and loss modulus obtained from experimental tests on the same coordinate system. The inverted U-shaped Cole–Cole curve formed by the scattered points is approximated using a third-order polynomial, as follows:
  (16) $$E^{\prime \prime }=p_{1}{E^{\prime }}^{3}+p_{2}{E^{\prime }}^{2}+p_{3}E^{\prime }+p_{4}$$
  where $p_{1}$, $p_{2}$, $p_{3}$, and $p_{4}$ are the coefficients of the third-order polynomial. Their values can be determined by fitting this polynomial to the test points based on the principle of least mean squares (L-M-S).
(2) Based on the determined Cole–Cole curve using the polynomial, calculate ${\hat{E}}_{0}$, ${\hat{E}}_{\infty }$, $dE^{\prime \prime }/dE^{\prime }$, tanφ, and tanθ.
(3) Calculate ${\hat{\alpha }}^{\prime }$ and ${\hat{\beta }}^{\prime }$ using Equation (10) and determine the peak point of the Cole–Cole curve $\left(E^{\prime }\left(\omega_{n}\right),E^{\prime \prime }\left(\omega_{n}\right)\right)$. Substitute this point into Equation (17) to check if ${\left(\omega_{n}\tau \right)}^{{\hat{\alpha }}^{’}}$ is smaller than 1:
(17) $${\left(\omega_{n}\tau \right)}^{{\hat{\alpha }}^{\prime }}=\frac{\Gamma \left(1+{\hat{\beta }}^{\prime }\right)}{2\Gamma \left(2+{\hat{\beta }}^{\prime }\right)}\cos\frac{{\hat{\alpha }}^{\prime }\pi }{2}$$
(4) Use the GA to calculate $\hat{\alpha }$ and $\hat{\beta }$. If ${\left(\omega_{n}\tau \right)}^{{\hat{\alpha }}^{’}}<1$, the objective function can be written as Equation (18):
(18) $$\begin{array}{cc} \min J_{1}= & \left|E^{\prime }\left(\omega_{n}\right)-\frac{{\hat{E}}_{0}}{E^{\prime \prime }\left(\omega_{n}\right)}-\frac{1}{2}\tan\frac{\alpha \pi }{2}-\frac{3}{2}\cot\frac{\alpha \pi }{2}\right|+\\ \; & \left|{\hat{E}}_{\infty }-\frac{E^{\prime }\left(\omega_{n}\right)}{E^{\prime \prime }\left(\omega_{n}\right)}-\frac{4\Gamma (2+\beta )\Gamma (\beta )}{\Gamma^{2}\left(1+\beta \right)}-\frac{3}{2}\right|\cot\frac{\alpha \pi }{2}+\frac{1}{2}\tan\frac{\alpha \pi }{2} \end{array}$$
  If ${\left(\omega_{n}\tau \right)}^{{\hat{\alpha }}^{’}}>1$, the objective function can be written as Equation (19).
(19) $$\begin{array}{c} \begin{array}{cc} \min J_{2}= & |E^{\prime }\left(\omega_{n}\right)-\frac{{\hat{E}}_{0}}{E^{\prime \prime }\left(\omega_{n}\right)}-\frac{\Gamma^{\beta }\left(1+\beta \right){\left(1+\beta \right)}^{\beta }}{\Gamma^{\beta }\left(\beta \right)\beta^{\beta }}\cos^{-\beta -1}\frac{\alpha \beta \pi }{2}\\ \; & \times \cos^{\beta }\frac{\alpha (1+\beta )\pi }{2}+\beta -\tan^{-1}\frac{\alpha \beta \pi }{2}-\beta \tan\frac{\alpha (1+\beta )\pi }{2}|\\ \; & +\left|{\hat{E}}_{\infty }-\frac{E^{\prime }\left(\omega_{n}\right)}{E^{\prime \prime }\left(\omega_{n}\right)}-{\left(1+\beta \right)}^{-1}\tan\alpha \beta \pi /2-\beta \tan\frac{\alpha (1+\beta )\pi }{2}\right| \end{array} \end{array}$$
(5) Based on the calculated ${\hat{E}}_{0}$, ${\hat{E}}_{\infty }$, $\hat{\alpha }$, and $\hat{\beta }$, determine the optimization interval and apply the GA again to optimize the temperature-independent parameters ($E_{0}$, $E_{\infty }$, α, and β) based on the objective function, as shown below:
  (20) $$\min J=\sum_{i}\sqrt{{\left(E^{\prime }-{E^{\prime }}_{\exp}\right)}^{2}+{\left(E^{\prime \prime }-{E^{\prime \prime }}_{\exp}\right)}^{2}}$$
  where ${E^{\prime }}_{\exp}$ and ${E^{\prime \prime }}_{\exp}$ represent the experimental storage modulus and loss modulus, respectively. The expressions of $E^{\prime }$ and $E^{\prime \prime }$ are presented in Appendix C for Equations (A3) and (A12), respectively.
(6) Based on the optimized values of the four parameters ($E_{0}$, $E_{\infty }$, α, and β), apply the GA again to calculate the relaxation time ($\tau_{i}$) at each temperature. Based on the WLF equation, calculate the shift factor $\alpha_{T}\left(T\right)$ at each test temperature with respect to the reference temperature (Tr = 30 ℃). $\alpha_{T}\left(T\right)$ defines the relationship between $\tau_{i}$ and $\tau_{ref}$, as shown below:
  (21) $$\mathrm{lg}\alpha_{T}(T)=\mathrm{lg}\tau_{i}-\mathrm{lg}\tau_{ref}$$
  According to the shift factors $\alpha_{T}(T)$ at different temperatures, the reduced frequency can be calculated based on Equation (22), as shown below:
  (22) $$\mathrm{lg}\omega_{red}=\mathrm{lg}\omega +\mathrm{lg}\alpha_{T}(T)$$

By shifting the experimental data at different temperatures, construct the master curve with a wider frequency range at the reference temperature. Additionally, input the parameters ($E_{0}$, $E_{\infty }$, α, β, and $\tau_{i}$) obtained through inverse analysis into the H–N model to calculate the $E^{\prime }$ and $E^{\prime \prime }$ at different temperatures and plot their master curves. Finally, compare the experimental data-based master curves with those constructed using the optimized parameters.

## 3. Results and Discussion

### 3.1. Case Study on the Optimization of the 2S2P1D Model

In this case study, dynamic test data of asphalt were utilized for optimizing the parameters of the 2S2P1D model. The dynamic shear rheometer (DSR) was employed to conduct tests at five temperatures (ranging from 0 to 40 ℃) and frequencies from 0.1 Hz to 100 Hz.

#### 3.1.1. Parameter Optimization Independent of the WLF Function

To optimize the parameters, the experimental data of the dynamic modulus $\left|G^{*}\right|$ were read, and $G_{0}$, $G_{g}$, k, h, α, β and five different relaxation times ($\tau_{i}$) corresponding to five temperatures were used as the unknowns. Then, the model parameters were optimized using the GA. According to the parameters’ physical meaning, the following constraints should be satisfied:

(23) $$\left\{\begin{array}{l} G_{0}-G_{g}<0\\ k-h<0 \end{array}\right.$$

(24) $$\min\;I={\left\|\frac{1}{n}\sum_{i=1}^{n}\frac{{\left|G^{*}\right|}_{i}-{\left|G^{*}\right|}_{\exp_{i}}}{{\left|G^{*}\right|}_{\exp_{i}}}\right\|}_{2}+{\left\|\frac{1}{n}\sum_{i=1}^{n}\frac{\delta_{i}-\delta_{\exp_{i}}}{\delta_{\exp_{i}}}\right\|}_{2}$$

These constraints facilitate the optimization process of the GA. The optimized values were obtained by minimizing the objective function defined in Equation (24), where ${\left|G^{*}\right|}_{\exp}$ and $\delta_{\exp}$ represent the experimental dynamic modulus and phase angle, respectively. The expressions for the dynamic modulus ($\left|G^{*}\right|$) and phase angle (δ) are given by Equations (4) and (8), respectively.

The parameter values for the GA solver are shown in Table 1. The fitted values of the parameters for the 2S2P1D model are presented in Table 2. Furthermore, the values were further optimized using the L–M algorithm, as shown in Table 3.

**Figure 4:**
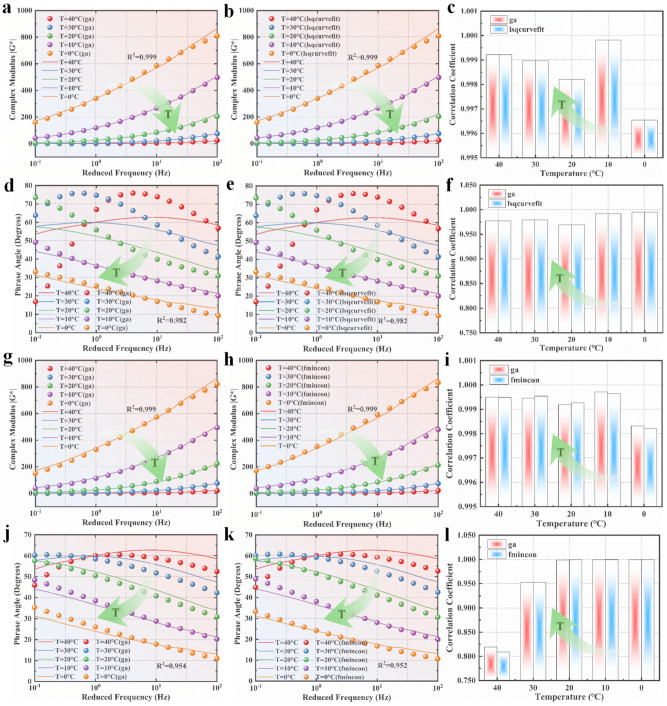
Experimental and fitted results for the 2S2P1D model: (**a**) dynamic modulus master curves fitted using the GA through Method 1; (**b**) dynamic modulus master curves fitted using both the genetic and L−M algorithms through Method 1; (**c**) fitting coefficients of the phase angle through Method 1; (**d**) phase angle master curves fitted using the GA through Method 1; (**e**) phase angle master curves fitted by both the genetic and L−M algorithms through Method 1; (**f**) fitting coefficients of the phase angle through Method 1; (**g**) dynamic modulus master curves fitted using the GA through Method 2; (**h**) dynamic modulus master curves fitted by both the genetic and L−M algorithms through Method 2; (**i**) fitting coefficients of the phase angle through Method 2; (**j**) phase angle master curves fitted using the GA through Method 2; (**k**) phase angle master curves fitted using both the genetic and L−M algorithms through Method 2; (**l**) fitting coefficients of the phase angle through Method 2.

Using the results obtained by applying the GA as the initial values, the L–M algorithm was employed for further optimization. By substituting the parameter values obtained from the first and second optimizations into Equations (4) and (8), we calculated the fitted dynamic modulus and phase angles. Additionally, we calculated the correlation coefficients between the experimental data and the values obtained from the GA. Figure 4a,b,d,e illustrate the experimental data, the calculated data obtained from the GA, and the quadratic fitted on the same coordinate system. Figure 4c,f depict the correlation coefficients. The pseudocode for the implementation of the L–M algorithm in this paper can be found in Appendix B and the parameter values for the L–M algorithm solver are shown in Table A1.

The optimized values by inverse calculation based on the GA were taken as the initial values and input into the L–M algorithm for secondary fitting. The general pseudocode for implementing the L–M algorithm in this paper is provided in Appendix B. The optimized values were then used in the 2S2P1D model (Equation (4)) to obtain the calculated dynamic modulus and phase angle. Figure 4a,b,d,e present the experimental data and the corresponding calculated curves. Figure 4c,f display the correlation coefficients. The pseudocode for implementing the L–M algorithm in this paper is included in Appendix B. 

#### 3.1.2. Parameter Optimization Dependent on the WLF Function

The experimental data of the dynamic modulus ($\left|G^{*}\right|$) and phase angle (δ) were used as inputs, and the parameters $G_{0}$, $G_{g}$, k, h, α, β, $\tau_{i}$, $C_{1}$, $C_{2}$ and $T_{r}$ were considered for optimization. These parameters have specific physical interpretations, and the following relationships should hold:

(25) $$\left\{\begin{array}{l} G_{0}-G_{g}<0\\ k-h<0\\ C_{1}-C_{2}<0 \end{array}\right.$$

(26) $$\min\;I={\left\|\frac{1}{n}\sum_{i=1}^{n}\frac{{\left|G^{*}\right|}_{i}-{\left|G^{*}\right|}_{\exp_{i}}}{{\left|G^{*}\right|}_{\exp_{i}}}\right\|}_{2}+{\left\|\frac{1}{n}\sum_{i=1}^{n}\frac{\delta_{i}-\delta_{\exp_{i}}}{\delta_{\exp_{i}}}\right\|}_{2}$$

The constraints were applied, and the upper and lower bounds were set for the GA. The optimized parameter values were determined by minimizing the objective function defined in Equation (26). To avoid overfitting, the data were normalized. The GA was configured with the parameter values shown in Table 4. The optimization process took 136.74 s. The resulting optimized parameter values for the 2S2P1D model are presented in Table 5.

The secondary optimization is performed using the L–M algorithm, and the resulting fitted parameter values are shown in Table 6. The calculation process took 2.15 s. By substituting the optimized parameters obtained from the L–M algorithm into the 2S2P1D model, the dynamic modulus and phase angle are calculated. Figure 4g,h,j,k illustrate the experimental data and the corresponding calculated curves, while Figure 4i,l depict the correlation coefficients.

As depicted in Figure 4c,i, the correlation coefficient of the dynamic modulus shows that the accuracy of the parameters obtained from both the GA and the L–M algorithm improved with increasing temperature. This suggests that the 2S2P1D model is more suitable for describing the dynamic modulus of asphalt at higher temperatures. Moreover, the correlation coefficients between the calculated values from the fitted models and the experimental data were consistently above 0.998, regardless of whether single fitting or secondary fitting was performed. It is noteworthy that Figure 4i demonstrates an improvement in the secondary fitting compared to the results obtained solely from the GA as the temperature decreased. This highlights the accuracy and practicality of the GA in finding optimal solutions.

In Figure 4f,l, the correlation coefficient of the phase angle reveals an enhancement in the accuracy of the fitting results obtained from both the GA and the L–M algorithm as the temperature decreased. This indicates that the 2S2P1D model provides more precise descriptions of the phase angle of asphalt under lower temperature conditions. The correlation coefficients between the results obtained from both the single fitting and secondary fitting and the experimental data are above 0.820. However, as the temperature decreased, the correlation coefficient of the secondary fitting gradually approached 1. In contrast to the dynamic modulus, the utilization of the second-order fitting consistently yielded better results in the phase angle correlation coefficient histogram compared to the use of the GA alone. This implies that the fitting results of the phase angle are more sensitive to the selection of the initial values when fitting the physical quantities of the viscoelastic properties of asphalt.

**Figure 5:**
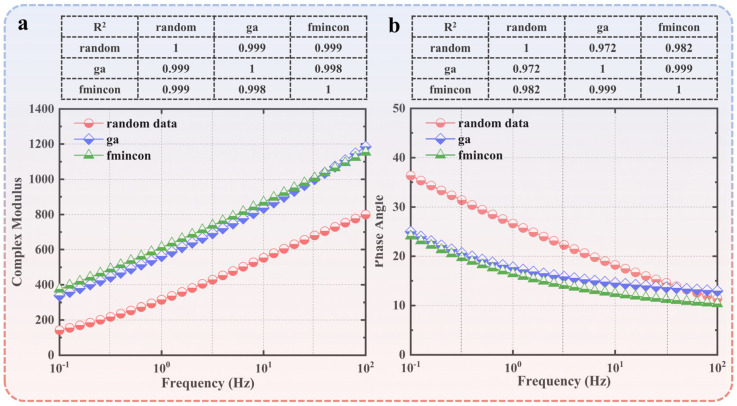
Calculated (**a**) dynamic modulus and (**b**) phase angle master curves based on the fitted 2S2P1D model.

In this study, the parameters obtained from the GA were incorporated into the 2S2P1D model. Numerical values within a predetermined range were generated and input into Equations (4) and (8) of the 2S2P1D model to calculate the corresponding dynamic modulus and phase angles. The calculated curves were then compared with the experimental data curves. Additionally, correlation coefficients were calculated between the experimental data and the values calculated using the GA and the secondary fitting using the L–M algorithm, as depicted in Figure 5. The results show that the trend of the modeled curves was consistent with the experimental data, indicating satisfactory fitting of the dynamic modulus and phase angle. The optimization methods successfully determined suitable values for the 2S2P1D model, which effectively describes the viscoelastic properties of asphalt.

### 3.2. Case Study on the Optimization of the H–N Model

The test data used in this case study were derived from the dynamic mechanical thermal analysis (DMTA) data of carbon-black-filled rubber (CBFR) NR100N550 [31]. By fitting the experimental data points of the Cole–Cole curve with a third-order polynomial function, the parameters (α, β, and γ) of the polynomial function (see Equation (17)) could be determined, as shown in Table 7. 

By substituting the experimental data of $E^{\prime }$ into the polynomial, the corresponding E″ could be obtained [10]. Based on Equation (17), setting $E^{\prime \prime }\;$= 0, the corresponding two estimated $E^{\prime }$ values, which correspond to ${\hat{E}}_{0}$ and ${\hat{E}}_{\infty }$, could be obtained. With further calculations of the derivative values of $dE^{\prime \prime }/dE^{\prime }$ at ${\hat{E}}_{0}$ and ${\hat{E}}_{\infty }$, the corresponding values of tanφ and tanθ could be derived. The results are presented in Table 8.

According to Equation (11), the estimations were as follows: ${\hat{\alpha }}^{\prime }$ = 0.564 and ${\hat{\beta }}^{\prime }$ = 0.400. Based on the peak point of the Cole–Cole curve $\left(E^{\prime }\left(\omega_{n}\right),E^{\prime \prime }\left(\omega_{n}\right)\right)$ = (1.746 × 10^3^, 9.370 × 10^2^). Substitute ${\hat{\alpha }}^{\prime }$ and ${\hat{\beta }}^{\prime }$ into Equation (17) to check whether ${\left(\omega_{n}\tau \right)}^{{\hat{\alpha }}^{\prime }}<1$. Using Equation (12), the estimated values of $\hat{\alpha }$ and $\hat{\beta }$ were calculated by optimizing the objective function with the GA, as shown in Equation (18). The lower limit of the optimization interval was $[{\hat{\alpha }}^{\prime }{\hat{\beta }}^{\prime }]\left(1-\Delta_{1}\right)$, and the upper limit was $[{\hat{\alpha }}^{\prime }{\hat{\beta }}^{\prime }]\left(1+\Delta_{1}\right)$. The calculation results are $\hat{\alpha }$ = 0.606 and $\hat{\beta }$ = 0.502. With the obtained ${\hat{E}}_{0}$, ${\hat{E}}_{\infty }$, $\hat{\alpha }$, and $\hat{\beta }$, the optimization interval was determined, and the objective function of Equation (20) was used. Considering the efficiency and calculation accuracy, the series order, *k*, in the objective function was set to five. Subsequently, the temperature-independent parameters ($E_{0}$, $E_{\infty }$, α, and β) were recalculated using the GA. The lower limit of the optimization interval was $[{\hat{E}}_{0}{\hat{E}}_{\infty }\hat{\alpha }\hat{\beta }]\left(1-\Delta_{2}\right)$, and the upper limit was $[{\hat{E}}_{0}{\hat{E}}_{\infty }\hat{\alpha }\hat{\beta }]\left(1+\Delta_{2}\right)$. The $\Delta_{1}$ and $\Delta_{2}$ were used to compensate for the rounding and fitting errors [10]. The obtained $E_{0}$, $E_{\infty }$, α , and β are presented in Table 9.

Using the initial optimized values obtained with the GA, a secondary optimization was performed using the L–M algorithm. The optimized values are shown in Table 10.

Based on the optimized temperature-independent parameters, as shown in Table 10, the relaxation times at different temperatures ($\tau_{i}$) were calculated, and the results are presented in Table 11.

**Figure 6:**
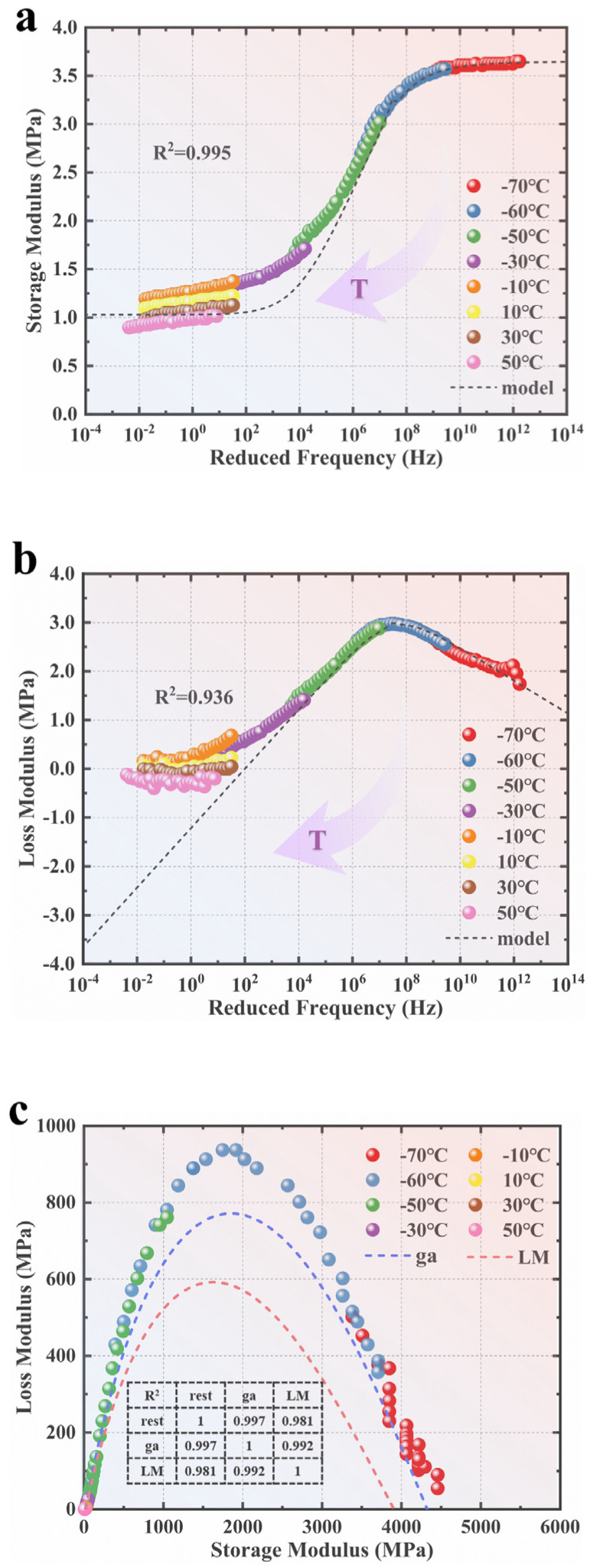
Fitted curves using the optimized H–N model: (**a**) master curves of $E^{\prime }$; (**b**) master curves of $E^{\prime \prime }$; (**c**) Cole−Cole curves.

A reference temperature $T_{r}=30\;℃$ was adopted. Based on the optimized $\tau_{i}$, the shift factors $\alpha_{T}\left(T\right)$ at each temperature and the reduced frequency $\omega_{red}$ could be calculated with Equations (21) and (22), respectively. Consequently, the experimental $E^{\prime }$ and $E^{\prime \prime }$ data at different temperatures could be plotted at the reference temperature. Additionally, the H–N model can be employed to construct master curves based on the optimized parameters. Figure 6a,b illustrate these master curves. Furthermore, the parameters obtained using the GA and the L–M algorithm were back-calculated and plotted on the same coordinate system as the experimental data to form a Cole–Cole curve. A comparison was made to evaluate the fitting effect, as shown in Figure 6c.

τ is an internal time index that characterizes the relaxation phenomena of materials and reflects their viscoelastic nature when subjected to external forces of similar time magnitude. Temperature has a significant effect on τ, thereby affecting the viscoelastic behavior of polymer materials. The Cole–Cole curve, which represents the relationship between the storage modulus and the loss modulus, is independent of temperature and unaffected by τ. Nevertheless, its evaluation is crucial to capturing the viscoelastic characteristics of the material.

Figure 6a,b illustrate the storage modulus and loss modulus over a wide frequency range. It can be observed that the storage modulus ($E^{\prime }$) exhibited a gradual increase followed by a rapid increase before reaching a plateau as the frequency increased. Conversely, the loss modulus ($E^{\prime \prime }$) initially increased and then decreased as the frequency increased. During the optimization process, the relaxation time ($\tau_{i}$) was set to 1 (i.e., treating each temperature as its own reference temperature), and separately fitting the parameters of $E_{0}$, $E_{\infty }$, α, and β would result in fitting within a narrow frequency range, known as the frequency locking phenomenon. However, this narrow range fails to capture the complete viscoelastic properties of materials. To address this, it becomes necessary to shift the test data at different temperatures to construct master curves that span a broader frequency range. Hence, when fitting the relaxation time $\tau_{i}$ at a reference temperature using the experimental data, it is necessary to calculate the shift factors $\alpha_{T}(T)$ at each test temperature to the reference temperature. 

Figure 6c demonstrates that the accuracy of the results obtained using the L–M algorithm for the secondary fitting was slightly lower than that of the GA. This discrepancy could be attributed to the overfitting problem resulting from a large amount of data or the greater influence of initial values on the L–M algorithm. Inaccurate initial values may lead to significant deviations between the fitting results of the L–M algorithm and the actual data. Therefore, the advantage of the GA is highlighted, as it can determine the global optimal solution without relying on initial values. However, the results obtained using the GA are strongly influenced by the upper and lower bounds. Consequently, in this study, the shape characteristics and peak positions of the Cole–Cole curve were considered when setting the upper and lower bounds for the four parameters using the GA to ensure minimal error in the calculation results.

During the H–N model fitting process, this study initially employed the Cole–Cole curve to estimate the values of the four parameters and set the upper and lower bounds for the subsequent inversion using the GA. This approach facilitates the solution process and effectively reduces errors caused by experimental data variations, given the large amount of data involved.

### 3.3. Sensitivity Analysis of the Input Variables

**Figure 7:**
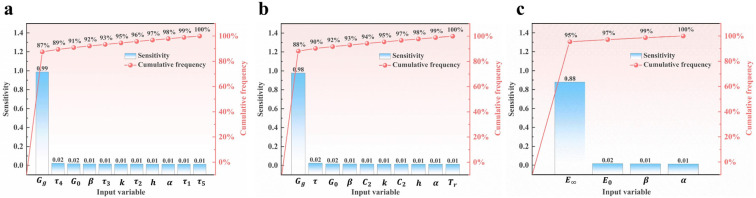
Pareto diagram of the sensitivity analysis of the input variables: (**a**) Pareto diagram of input variables in Method 1 of the 2S2P1D model; (**b**) Pareto diagram of input variables in Method 2 of the 2S2P1D model; (**c**) Pareto diagram of input variables in the H–N model.

A sensitivity analysis of the input variables of the two viscoelastic models, the 2S2P1D model and H–N model, was conducted, and a Pareto diagram was drawn, as shown in Figure 7. It can be found that the first-order sensitivity of $G_{g}$ and $E_{\infty }$ is the largest and far exceeds that of other variables, indicating that these two input variables have a greater influence on the objective function. Therefore, it is more necessary to set the upper and lower bounds of the input variables reasonably, which is helpful for reducing unnecessary computations, improving the efficiency of the optimization algorithm, thus searching for and finding the optimal solution more effectively.

## 4. Findings and Conclusions

This paper presented the development of optimization methods for the parameters of two widely used viscoelastic models: 2P2S1D and H–N models. The GA and L–M algorithm were utilized for parameter optimization. Based on the results of this study, the following findings can be summarized:

1. Two optimization methods were employed for the 2S2P1D model: one independent of the WLF equation utilizing the GA and one dependent on the WLF equation utilizing the L–M algorithm. A comparison of the fitting results revealed that the optimization method independent of the WLF equation exhibited higher accuracy in parameter fitting, while the method dependent on the WLF equation demonstrated slightly lower accuracy.
2. For the 2S2P1D model, the fitting accuracy of the parameters optimized using the GA could be further improved by secondary optimization using the L–M algorithm. This suggests that a combination of the GA and L–M algorithm improves the overall fitting accuracy.
3. The H–N model often encounters complex fractional-order derivatives during the GA fitting, resulting in a loss of physical meaning in the solution domain and optimization failure. To address this issue, the Cole–Cole curve was initially fitted with a third-order polynomial function, followed by further fitting using the GA with given upper and lower bounds. This improved method can increase the accuracy of the calculated results. However, the optimized values are significantly influenced by the experimental data, and it is challenging to improve the accuracy using the L–M algorithm. This may be closely related to the method used to collect the fitting data values.

## Figures and Tables

**Table 1:** Values of the parameters of the GA solver.

paretoFraction (-)	CrossoverFraction (-)	UseParallel (-)	PopulationSize (-)	Generations (-)	stallGenLimit (-)	TolFun (-)
0.3	0.8	true	2000	200	200	10^−10^

**Table 2:** Optimized parameters’ values based on the GA.

$G_{0}$ (MPa)	$G_{g}$ (MPa)	k (-)	h (-)	α (-)	β (-)	$\tau_{1}$ (-)	$\tau_{2}$ (-)	$\tau_{3}$ (-)	$\tau_{4}$ (-)	$\tau_{5}$ (-)
2.255	1.230 × 10^3^	0.367	0.349	9.99	0.325	1.879	1.409	1.487	1.983	1.935

**Table 3:** Optimized parameters’ values based on the L–M algorithm.

$G_{0}$ (MPa)	$G_{g}$(MPa)	k (-)	h (-)	α (-)	β (-)	$\tau_{1}$ (-)	$\tau_{2}$ (-)	$\tau_{3}$ (-)	$\tau_{4}$ (-)	$\tau_{5}$ (-)
0.176	1.062 × 10^3^	0.395	0.985	9.999	1.574	1.696	1.361	1.433	1.960	1.940

**Table 4:** Parameter values for the GA solver.

paretoFraction (-)	CrossoverFraction (-)	UseParallel (-)	PopulationSize (-)	Generations (-)	stallGenLimit (-)	TolFun (-)
0.3	0.8	true	500	200	200	10^−20^

**Table 5:** Optimized parameter values based on the GA.

$G_{0}$ (MPa)	$G_{g}$ (MPa)	k (-)	h (-)	α (-)	β (-)	τ (-)	$C_{1}$ (-)	$C_{2}$ (-)	$T_{r}$ (℃)
0.0943	1.247 × 10^3^	0.329	0.704	3.615	99.999	2.981	40.443	298.987	7.943 × 10^−5^

**Table 6:** Optimized parameters’ values based on the L–M algorithm.

$G_{0}$ (MPa)	$G_{g}$ (MPa)	k (-)	h (-)	α (-)	β (-)	τ (-)	$C_{1}$ (-)	$C_{2}$ (-)	$T_{r}$ (℃)
0.095	1.247 × 10^3^	0.321	0.691	3.309	100	2.143	40.100	298.458	0.445

**Table 7:** Fitted parameters of the third-order polynomial function.

$p_{1}$ (-)	$p_{2}$ (-)	$p_{3}$ (-)	$p_{4}$ (-)
4.204 × 10^−8^	−4.635 × 10^−4^	1.243	−27.113

**Table 8:** Estimated ${\hat{E}}_{0}$, ${\hat{E}}_{\infty }$ tan φ and tan θ.

${\hat{E}}_{0}$ (MPa)	${\hat{E}}_{\infty }$ (MPa)	tanφ (-)	tanθ (-)
15.678	4.515 × 10^3^	1.189	−0.378

**Table 9:** Optimized values based on the GA.

$E_{0}(\mathrm{MPa})$	$E_{\infty }(\mathrm{MPa})$	α (-)	β (-)
17.245	4.325 × 10^3^	0.546	0.545

**Table 10:** Optimized values using the secondary L–M algorithm.

$E_{0}(\mathrm{MPa})$	$E_{\infty }(\mathrm{MPa})$	α (-)	β (-)
18.970	3.906 × 10^3^	0.491	0.492

**Table 11:** Optimized relaxation times at different temperatures.

Temperature (℃)	−70	−60	−50	−30	−10	10	30	50
$\tau_{i}$ (-)	5.206 × 10^2^	1.975	0.663	0.616	10^−8^	10^−8^	10^−8^	2.390 × 10^−9^

## Data Availability

Data will be made available on request.

## Appendix A

The pseudocode of the GA.

**Procedure** **SGA**
**begin** Initialize P(0); t = 0; ** while** (t ≤ T) **do** **for** i = 1 to M **do** Evaluate fitness of P(t); ** end for** ** for** i = 1 to M **do** Select operation to P(t); ** end for** ** for** i = 1 to M/2 **do** Crossover operation to P(t); ** end for** ** for** i = 1 to M **do** Mutation operation to P(t); ** end for** ** for** i = 1 to M **do** P(t + 1) = P(t) ** end for** t = t + 1; ** end while** **end**

## Appendix B

Pseudocode of the L–M algorithm.

Target: to estimate p in the function x=f(p) with the given f(⋅) and observation vector x with noise.

Calculation steps:

(1) Choose initial point p_0_; control the constant for termination ε; calculate $\varepsilon_{0}=\left\|x-f(p_{0})\right\|$, set k = 0, $\lambda_{0}=10^{-3}$, and v = 10 (or another number greater than 1).
(2) Calculate the Jacobian matrix $J_{k}^{T}$, ${\bar{N}}_{k}=J_{k}^{T}J_{k}+\lambda_{k}I$, and construct the increment normal equation ${\bar{N}}_{k}\cdot \delta_{k}=J_{k}^{T}\varepsilon_{k}$.
(3) Solve the increment normal equation to obtain $\delta_{k}$:
  ① If $\left\|x-f(p_{k}+\delta_{k})\right\|<\varepsilon_{k}$, set $p_{k+1}=p_{k}+\delta_{k}$, and if $\left\|\delta_{k}\right\|<\varepsilon_{}$, stop the iteration and output the results. Otherwise, set $\lambda_{k+1}=\lambda_{k}/v$, and go to Step 2;
  ② If $\left\|x-f(p_{k}+\delta_{k})\right\|\geq \varepsilon_{k}$, set $\lambda_{k+1}=\lambda_{k}\cdot v$, re-solve the normal equation to obtain $\delta_{k}$, and return to Step 1.

Pseudocode:

**Table A1:** Parameter values for the L–M algorithm solver.

Algorithm (-)	TolFun (-)	MaxFunctionEvaluations (-)	MaxIterations (-)
sqp-legacy algorithm	1 × 10^−40^	1 × 10^4^	1 × 10^3^

**Input**: A vector function $f:R^{m}\to R^{n}$ with n≥m, a measurement vector $x\in R^{n}$ and an initial parameter estimate $p_{0}\in R^{m}$. **Output**: A vector $p^{+}\in R^{m}$ minimizing ${\left\|x-f(p)\right\|}^{2}$. **Algorithm**: **begin** k := 0; v := 2; and p := p_0_; A = J^T^J; $\varepsilon_{p}:=x-f(p)$; and g := J^T^$\varepsilon_{p}$; Stop := (${\left\|g\right\|}_{\infty }\leq \varepsilon_{1}$); $\mu :=\tau *\max_{i,\ldots ,m}(A_{ii})$; **while** (not stop) and (k < k_max_) k := k + 1; repeat Solve $(A+\mu I)\delta_{p}=g$; **if**($\left\|\delta_{p}\right\|\leq \varepsilon_{2}\left\|p\right\|$) stop := true; **else** p_new_ := p + $\delta_{p}$ $\rho :=({\left\|\varepsilon_{p}\right\|}^{2}-{\left\|x-f()p_{new}\right\|}^{2})/(\delta_{p}^{T}(\mu \delta_{p}^{}+g));$ **if** ρ>0 p = p_new_; A = J^T^J; $\varepsilon_{p}:=x-f(p)$; and g := J^T^$\varepsilon_{p}$; stop := (${\left\|g\right\|}_{\infty }\leq \varepsilon_{1}$) or (${\left\|\varepsilon_{p}\right\|}^{2}\leq \varepsilon_{3}$); $\mu :=\mu *\max(\frac{1}{3},1-{\left(2\rho -1\right)}^{3});v:=2;$ **else** μ:=μ*v;v:=2*v; **end if** **end if** **until** (ρ>0) or (stop) **end while** p^+^ := p; **end**

## Appendix C

Formula derivation.

(1) In the H–N model’s Cole–Cole domain, $\tan\varphi =\underset{\omega \to 0}{\lim}dE^{\prime \prime }\left(\omega \right)/dE^{\prime }\left(\omega \right)=\tan\alpha \pi /2$.
  When ω→0, $\left|i\omega \tau \right|<1$. The following series can be found in Hilfer [32]:
  (A1) $$\begin{array}{c} \begin{array}{c} \begin{array}{cc} \frac{1}{{\left[1+{\left(i\omega \tau \right)}^{\alpha }\right]}^{\beta }} & =\sum_{k=0}^{\infty }\frac{{\left(-1\right)}^{k}\Gamma \left(k+\beta \right)}{\Gamma (\beta )\Gamma (k+1)}{\left(i\omega \tau \right)}^{\alpha k}\\ \; & =\sum_{k=1}^{\infty }\frac{{\left(-1\right)}^{k}\Gamma \left(k+\beta \right)}{\Gamma (\beta )\Gamma (k+1)}{\left(i\omega \tau \right)}^{\alpha k}+1 \end{array} \end{array} \end{array}$$
  Substituting Equation (A1) into Equation (1), the H–N equation is displayed as:
  (A2) $$\begin{array}{c} \begin{array}{c} \begin{array}{c} \begin{array}{cc} E^{*}\left(\omega \right)= & E_{0}+\left(E_{0}-E_{\infty }\right)\sum_{k=1}^{\infty }\frac{{\left(-1\right)}^{k}\Gamma \left(k+\beta \right)}{\Gamma (\beta )\Gamma (k+1)}\cos\frac{\alpha k\pi }{2}{\left(\omega \tau \right)}^{\alpha k}\\ \; & +i\left(E_{0}-E_{\infty }\right)\sum_{k=1}^{\infty }\frac{{\left(-1\right)}^{k}\Gamma \left(k+\beta \right)}{\Gamma (\beta )\Gamma (k+1)}\sin\frac{\alpha k\pi }{2}{\left(\omega \tau \right)}^{\alpha k} \end{array} \end{array} \end{array} \end{array}$$
  The storage modulus $E^{\prime }\left(\omega \right)$ and loss modulus $E^{\prime \prime }\left(\omega \right)$ are displayed as:
  (A3) $$\begin{array}{l} E^{\prime }\left(\omega \right)=E_{0}+\left(E_{0}-E_{\infty }\right)\sum_{k=1}^{\infty }\frac{{\left(-1\right)}^{k}\Gamma \left(k+\beta \right)}{\Gamma \left(\beta \right)\Gamma \left(k+1\right)}\cos\frac{\alpha k\pi }{2}{\left(\omega \tau \right)}^{\alpha k}\\ E^{\prime \prime }\left(\omega \right)=\left(E_{0}-E_{\infty }\right)\sum_{k=1}^{\infty }\frac{{\left(-1\right)}^{k}\Gamma \left(k+\beta \right)}{\Gamma \left(\beta \right)\Gamma \left(k+1\right)}\sin\frac{\alpha k\pi }{2}{\left(\omega \tau \right)}^{\alpha k} \end{array}$$
  (A4) $$\begin{array}{l} \frac{dE^{\prime }\left(\omega \right)}{d\omega }=\left(E_{0}-E_{\infty }\right)\sum_{k=1}^{\infty }\frac{{\left(-1\right)}^{k}\Gamma \left(k+\beta \right)}{\Gamma \left(\beta \right)\Gamma \left(k+1\right)}\cos\frac{\alpha k\pi }{2}\alpha k\tau^{\alpha k}\omega^{\alpha k-1}\\ \frac{dE^{\prime \prime }\left(\omega \right)}{d\omega }=\left(E_{0}-E_{\infty }\right)\sum_{k=1}^{\infty }\frac{{\left(-1\right)}^{k}\Gamma \left(k+\beta \right)}{\Gamma \left(\beta \right)\Gamma \left(k+1\right)}\sin\frac{\alpha k\pi }{2}\alpha k\tau^{\alpha k}\omega^{\alpha k-1} \end{array}$$
  Let N be the maximum integer that satisfies αN<1. When ω→0, Equation (A5) can be approximated as:
  (A5) $$\begin{array}{l} \underset{\omega \to 0}{\lim}\frac{dE^{\prime }\left(\omega \right)}{d\omega }=\underset{\omega \to 0}{\lim}\left(E_{0}-E_{\infty }\right)\sum_{k=1}^{N}\frac{{\left(-1\right)}^{k}\Gamma \left(k+\beta \right)}{\Gamma \left(\beta \right)\Gamma \left(k+1\right)}\cos\frac{\alpha k\pi }{2}\alpha k\tau^{\alpha k}\omega^{\alpha k-1}\\ \underset{\omega \to 0}{\lim}\frac{dE^{\prime \prime }\left(\omega \right)}{d\omega }=\underset{\omega \to 0}{\lim}\left(E_{0}-E_{\infty }\right)\sum_{k=1}^{N}\frac{{\left(-1\right)}^{k}\Gamma \left(k+\beta \right)}{\Gamma \left(\beta \right)\Gamma \left(k+1\right)}\sin\frac{\alpha k\pi }{2}\alpha k\tau^{\alpha k}\omega^{\alpha k-1}\; \end{array}$$
  So, we can obtain:
  (A6) $$\begin{array}{cc} \lim_{\omega \to 0}\frac{dE^{\prime \prime }\left(\omega \right)}{dE^{\prime }\left(\omega \right)} & =\lim_{\omega \to 0}\frac{\frac{dE^{\prime \prime }\left(\omega \right)}{d\omega }}{\frac{dE^{\prime }\left(\omega \right)}{d\omega }}\\ \; & =\lim_{\omega \to 0}\frac{\sum_{k=1}^{N}\frac{{\left(-1\right)}^{k}\Gamma \left(k+\beta \right)}{\Gamma (k+1)}\sin\frac{\alpha k\pi }{2}k\tau^{\alpha k}\omega^{\alpha k-1}}{\sum_{k=1}^{N}\frac{{\left(-1\right)}^{k}\Gamma \left(k+\beta \right)}{\Gamma (k+1)}\cos\frac{\alpha k\pi }{2}k\tau^{\alpha k}\omega^{\alpha k-1}} \end{array}$$
  (A7) $$\tan\varphi =\underset{\omega \to 0}{\lim}\frac{dE^{\prime \prime }\left(\omega \right)}{dE^{\prime }\left(\omega \right)}=\underset{\omega \to 0}{\lim}\frac{\Sigma_{k=1}^{N}\frac{{\left(-1\right)}^{k}\Gamma \left(k+\beta \right)}{\Gamma \left(k+1\right)}\sin\frac{\alpha k\pi }{2}k\tau^{\alpha k}\omega^{\alpha \left(k-1\right)}}{\Sigma_{k=1}^{N}\frac{{\left(-1\right)}^{k}\Gamma \left(k+\beta \right)}{\Gamma \left(k+1\right)}\cos\frac{\alpha k\pi }{2}k\tau^{\alpha k}\omega^{\alpha \left(k-1\right)}}$$
  For any positive integer N, Equation (A7) is equal to:
  (A8) $$\tan\varphi =\underset{\omega \to 0}{\lim}\frac{dE^{\prime \prime }\left(\omega \right)}{dE^{\prime }\left(\omega \right)}=\tan\frac{\alpha \pi }{2}$$
(2) In the H–N model’s Cole–Cole domain [32], $\tan\theta =\underset{\omega \to \infty }{\lim}dE^{\prime \prime }\left(\omega \right)/dE^{\prime }\left(\omega \right)=-\tan\alpha \beta \pi /2.$
  When $\omega \to \infty ,\left|i\omega \tau \right|>1$ [32]:
  (A9) $$\frac{1}{{\left[1+{\left(i\omega \tau \right)}^{\alpha }\right]}^{\beta }}=\sum_{k=0}^{\infty }\frac{{\left(-1\right)}^{k}\Gamma \left(k+\beta \right)}{\Gamma \left(\beta \right)\Gamma \left(k+1\right)}{\left(i\omega \tau \right)}^{-\alpha \left(k+\beta \right)}$$
  Applying Equation (A9), the H–N model’s equation can be expanded in series as:
  (A10) $$E^{*}\left(\omega \right)=E_{\infty }+\left(E_{0}-E_{\infty }\right)\sum_{k=0}^{\infty }\frac{{\left(-1\right)}^{k}\Gamma \left(k+\beta \right)}{\Gamma \left(\beta \right)\Gamma \left(k+1\right)}{\left(i\omega \tau \right)}^{-\alpha \left(k+\beta \right)}$$
  (A11) $$\begin{array}{cc} E^{*}\left(\omega ,r\right)= & E_{\infty }+\left(E_{0}-E_{\infty }\right)\\ \; & \times \sum_{k=0}^{\infty }\frac{{\left(-1\right)}^{k}\Gamma \left(k+\beta \right)}{\Gamma (\beta )\Gamma (k+1)}\cos\frac{\alpha (k+\beta )\pi }{2}{\left(\omega \tau \right)}^{-\alpha (k+\beta )}\\ \; & -i\left(E_{0}-E_{\infty }\right)\\ \; & \times \sum_{k=0}^{\infty }\frac{{\left(-1\right)}^{k}\Gamma \left(k+\beta \right)}{\Gamma (\beta )\Gamma (k+1)}\sin\frac{\alpha (k+\beta )\pi }{2}{\left(\omega \tau \right)}^{-\alpha (k+\beta )} \end{array}$$
  The storage modulus $E^{\prime }\left(\omega \right)$ and loss modulus $E^{\prime \prime }\left(\omega \right)$ should be:
  (A12) $$\begin{array}{l} E^{\prime }\left(\omega \right)=E_{\infty }+\left(E_{0}-E_{\infty }\right)\sum_{k=0}^{\infty }\frac{{\left(-1\right)}^{k}\Gamma \left(k+\beta \right)}{\Gamma \left(\beta \right)\Gamma \left(k+1\right)}\cos\frac{\alpha \left(k+\beta \right)\pi }{2}{\left(\omega \tau \right)}^{-\alpha \left(k+\beta \right)}\\ E^{\prime \prime }\left(\omega \right)=-\left(E_{0}-E_{\infty }\right)\sum_{k=0}^{\infty }\frac{{\left(-1\right)}^{k}\Gamma \left(k+\beta \right)}{\Gamma \left(\beta \right)\Gamma \left(k+1\right)}\sin\frac{\alpha \left(k+\beta \right)\pi }{2}{\left(\omega \tau \right)}^{-\alpha \left(k+\beta \right)} \end{array}$$
  (A13) $$\begin{array}{cc} \frac{dE^{\prime }\left(\omega \right)}{d\omega }= & \left(E_{0}-E_{\infty }\right)\sum_{k=0}^{\infty }\frac{{\left(-1\right)}^{k+1}\Gamma \left(k+\beta \right)}{\Gamma \left(\beta \right)\Gamma \left(k+1\right)}\cos\frac{\alpha \left(k+\beta \right)\pi }{2}\\ & \times \alpha \left(k+\beta \right)\tau^{-\alpha \left(k+\beta \right)}\omega^{-\alpha \left(k+\beta \right)-1}\\ \frac{dE^{\prime \prime }\left(\omega \right)}{d\omega }= & \left(E_{0}-E_{\infty }\right)\sum_{k=0}^{\infty }\frac{{\left(-1\right)}^{k}\Gamma \left(k+\beta \right)}{\Gamma \left(\beta \right)\Gamma \left(k+1\right)}\sin\frac{\alpha \left(k+\beta \right)\pi }{2}\\ & \times \alpha \left(k+\beta \right)\tau^{-\alpha \left(k+\beta \right)}\omega^{-\alpha \left(k+\beta \right)-1} \end{array}$$
  Substituting *t* for 1/ω, Equation (A13) can be rewritten as:
  (A14) $$\begin{array}{cc} \frac{dE^{\prime }\left(\omega \right)}{d\omega }= & \left(E_{0}-E_{\infty }\right)\sum_{k=0}^{\infty }\frac{{\left(-1\right)}^{k+1}\Gamma \left(k+\beta \right)}{\Gamma \left(\beta \right)\Gamma \left(k+1\right)}\cos\frac{\alpha \left(k+\beta \right)\pi }{2}\\ & \times \alpha \left(k+\beta \right)\tau^{-\alpha \left(k+\beta \right)}t^{\alpha \left(k+\beta \right)+1}\\ \frac{dE^{\prime \prime }\left(\omega \right)}{d\omega }= & \left(E_{0}-E_{\infty }\right)\sum_{k=0}^{\infty }\frac{{\left(-1\right)}^{k}\Gamma \left(k+\beta \right)}{\Gamma \left(\beta \right)\Gamma \left(k+1\right)}\sin\frac{\alpha \left(k+\beta \right)\pi }{2}\\ & \times \alpha \left(k+\beta \right)\tau^{-\alpha \left(k+\beta \right)}t^{\alpha \left(k+\beta \right)+1} \end{array}$$
  Then, tanθ can be attained as:
  (A15) $$\begin{array}{cc} \tan\theta & =\lim_{\omega \to \infty }\frac{dE^{\prime \prime }\left(\omega \right)}{dE^{\prime }\left(\omega \right)}=\lim_{\omega \to 0}\frac{\frac{dE^{\prime \prime }\left(\omega \right)}{d\omega }}{\frac{dE^{\prime }\left(\omega \right)}{d\omega }}\\ \; & =\lim_{t\to 0}\frac{\sum_{k=0}^{\infty }\frac{{\left(-1\right)}^{k}\Gamma \left(k+\beta \right)}{\Gamma (\beta )\Gamma (k+1)}\sin\frac{\alpha (k+\beta )\pi }{2}\alpha \left(k+\beta \right)\tau^{-\alpha (k+\beta )}t^{\alpha (k+\beta )+1}}{\sum_{k=0}^{\infty }\frac{{\left(-1\right)}^{k+1}\Gamma \left(k+\beta \right)}{\Gamma (\beta )\Gamma (k+1)}\cos\frac{\alpha (k+\beta )\pi }{2}\alpha \left(k+\beta \right)\tau^{-\alpha (k+\beta )}t^{\alpha (k+\beta )+1}}\\ \; & =\lim_{t\to 0}\frac{\sum_{k=0}^{\infty }\frac{{\left(-1\right)}^{k}\Gamma \left(k+\beta \right)}{\Gamma (k+1)}\sin\frac{\alpha (k+\beta )\pi }{2}\left(k+\beta \right)\tau^{-\alpha k}t^{\alpha k}}{\sum_{k=0}^{\infty }\frac{{\left(-1\right)}^{k+1}\Gamma \left(k+\beta \right)}{\Gamma (k+1)}\cos\frac{\alpha (k+\beta )\pi }{2}\left(k+\beta \right)\tau^{-\alpha k}t^{\alpha k}}\\ \; & =-\tan\frac{\alpha \beta \pi }{2} \end{array}$$
